# Additional Risk Factors Lead to a Measurable Inflammatory Response in Stage I Endometrial Cancer—A Prospective Multicentric Observational Study

**DOI:** 10.1007/s13193-025-02228-5

**Published:** 2025-02-24

**Authors:** Carlo Ronsini, Irene Iavarone, Maria Giovanna Vastarella, Luigi Della Corte, Giada Andreoli, Giuseppe Bifulco, Luigi Cobellis, Pasquale de Franciscis

**Affiliations:** 1 Department of Woman, Child and General and Specialized Surgery, University of Campania “Luigi Vanvitelli”, Largo Madonna Delle Grazie, 1-80138 Naples, Italy; 2 Department of Neuroscience, Reproductive Sciences and Dentistry, School of Medicine, University of Naples, 80138 Naples, Italy

**Keywords:** Endometrial carcinoma, Myometrial infiltration, Lymphovascular space invasion (LVSI), Inflammation indices, Diagnosis

## Abstract

The objective of the study is to explore the correlation between inflammation indices (NLR, MLR, PLR) and prognostic factors (myometrial infiltration, LVSI, grading) in FIGO 2023 stage I endometrial carcinoma. From August 2023 to March 2024, a prospective study was conducted on 163 women diagnosed with stage I endometrial cancer. The research methods were established a priori and authorized through evaluation by the Ethics Committee of the individual centers (IRB 30661/2022 of 31/03/2022). The study was then registered on the clinicaltrials.gov platform under NCT05657483. Blood samples were collected preoperatively to measure the neutrophil-to-lymphocyte ratio (NLR), monocyte-to-lymphocyte ratio (MLR), and platelet-to-lymphocyte ratio (PLR). Histopathological data on myometrial infiltration, LVSI, and grading were also analyzed. NLR values were 2.07, 2.35, and 2.68 for no infiltration, < 50%, and ≥ 50% myometrial infiltration, respectively (*p* = 0.033). MLR values were 0.20, 0.23, and 0.26 for the same categories (*p* = 0.029). PLR values were 119, 140, and 146 (*p* = 0.043). For LVSI, NLR was 2.17 in negative and 3.23 in diffuse (*p* = 0.010), while MLR showed 0.20 vs 0.24 (*p* = 0.054), and PLR showed 125 vs 141 (*p* = 0.033). Multivariate analysis indicated myometrial infiltration had the strongest correlation with inflammation indices (beta 0.07, CI 95% 0.01–0.13, *p* = 0.041). Inflammation indices (NLR, MLR, PLR) significantly correlate with myometrial infiltration, LVSI positivity, and higher grading in early-stage endometrial carcinoma, with myometrial infiltration showing the strongest association. These findings suggest that inflammation indices could aid in the prognostic evaluation of endometrial carcinoma. Further research is needed to understand the prognostic implications fully.

## Introduction

Recently, the FIGO 2023 classification has reshaped the concept of stage I endometrial carcinoma [1]. More emphasis has been placed on factors such as histotype, myometrial infiltration, and lymphovascular space invasion (LVSI) [2, 3]. This staging attempts to give prognostic significance to microscopic factors of local infiltration and tumor aggressiveness. On the other hand, it is known in the literature how tumor progression may be accompanied by a response from the patient’s immune system. In numerous solid tumors, this response has been shown to correlate with diagnostic and prognostic factors [4–6]. Even in endometrial carcinoma, alterations in indices such as the neutrophil-to-lymphocyte ratio (NLR), monocyte-to-lymphocyte ratio (MLR), and platelet-to-lymphocyte ratio (PLR) have already demonstrated predictive and prognostic significance [7]. However, it is easy to assume that the inflammatory response, and thus the alteration of these inflammation indices, is determined by local tumor progression. It is reasonable to think that the deepening of the tumor in the myometrial tissue and the reaching of the LVSI represents a reason for contact between the tumor and the patient, such as to trigger an inflammatory response. However, it is not entirely clear what progression factor most influences the subject’s immune response. A better understanding of the molecular and microscopic mechanisms of tumor progression is the key to unlocking new therapeutic horizons. Recently, introducing dostarlimab into clinical practice has been an effective way of harnessing the host immune system through immunotherapy [8]. In this setting, our study aims to explore further alterations in inflammation indices corresponding to factors suggestive of local disease progression, such as myometrial infiltration, LVSI, and grading. We conducted a prospective study or observation on stage I endometrial carcinomas for this.

## Materials and Methods

### Ethical or Institutional Review Board Approval

The research methods were established a priori and authorized through evaluation by the Ethics Committee of the individual centers (IRB 30661/2022 of 31/03/2022). The study was then registered on the clinicaltrials.gov platform under NCT05657483.

### Study Design

Between August 2023 and March 2024, all women referred to AOU Vanvitelli, and Policlinico Federico II in Naples, Italy, with FIGO 2023 stage I endometrial cancer who met all the inclusion criteria and none of the exclusion criteria were prospectively enrolled in the study with no randomization. All patients freely participated in the study and signed an informed consent.

The inclusion criteria were age ≥ 18 years, histological diagnosis of endometrial cancer, and patients undergoing surgical staging of pathology, with complete anatomopathological information obtainable. The exclusion criteria were patients with chronic inflammatory diseases (inflammatory bowel disease, rheumatological pathologies, autoimmunity diseases), patients with an additional synchronous oncological diagnosis or within the previous 3 years, patients with corticosteroid overproduction disorders, and patients on steroid therapy within the last 30 days before recruitment.

### Data Collection

All patients enrolled in the study underwent blood sampling the day before surgery.

The data collected preoperatively, immediately after previous surgery, were blood counts of neutrophils, leukocytes, monocytes, platelets, and eosinophils. The haematochemical parameters were then expressed as neutrophil-to-lymphocyte ratio (NLR), monocyte-to-lymphocyte ratio (MLR), and platelet-to-lymphocyte ratio (PLR).

The ratio was defined as absolute cell count divided by absolute lymphocyte count. All patients then underwent surgery according to international standards of care [9, 10].

We also collected histopathological data on myometrial infiltration, grading, histotype, microsatellite stability, and LVSI. Myometrial Infiltration was expressed as absent (no infiltration, < 50%, and ≥ 50%). LVSI was expressed as a semiquantitative evaluation. Negative or focal involvement was considered negative. Only diffuse infiltration was assessed as positive [11–13]. In the case of post-surgical factors suggestive of stages higher than I, such as lymph node positivity, the patient was excluded from the study analysis. Negative or focal involvement was considered negative. All the parameters collected were kept in unique Clinical Registration Forms (CRF) and digitized in an electronic database on Excel software anonymously, under the responsibility of the Principal Investigator. They will be destroyed after the results are published.

### Statistic Analysis

The nominal variables were expressed as absolute frequency and percentages and compared using Fisher’s exact [14] and chi-square tests [15]. Continuous variables were expressed as median and interquartile range and compared using the Wilcoxon test [16]. The comparison of variables to more than two independent groups was conducted via Kruskal–Wallis [17].

The null hypothesis of our study was that there was no difference in the mean values of NLR, MLR, PLR, between patients with no myometrial infiltration, < 50%, and ≥ 50% (H0: μ1 = μ2 = μ3;). Secondary outcomes were the same as negative or focal LVSI (negative) or positive (H0: μ1 = μ2; H1: μ1-μ2 ≠ 0 two-sides) and grading (H0: μ). We conducted a multivariate linear regression to demonstrate a correction between the parameters examined and the alterations in inflammation indices regression [18].

The significance of the model used was assessed using the maximum likelihood method [19]. The distribution of the continuous variables for the individual parameters of the reference outcome was graphed in boxplots. The statistical significance level was set at 0.05.

All statistical investigations were performed using R software and R Studio vers. 2023.12.1 + 402.

### Risk of Bias

Multivariate regression studies were conducted with a combination of all variables present to minimize confounders. The individual models thus obtained were compared using adjusted R2 and Bayesian information criteria (BIC). The best model was chosen based on the lowest expressed value of BIC. Data analysis was conducted first by CR and then by Blinding by LDC, who was unaware of the study’s objective. No Missing data were present in the outcomes of interest.

## Results

Between August 2023 and March 2024, 192 women were enrolled in the study. After acquiring anatomopathological data, 29 patients were excluded (17 lymph node positivity, 6 stage IIB, 3 stage IIC, 3 stage IVB). A total of 163 women were included in the analysis. All enrolled patients were evaluated for neutrophils, lymphocytes, monocytes, eosinophils, and platelets. Clinics and demographic data were collected. The mean age was 62 years, and the mean BMI was 26; 84% of patients showed microsatellite stability (MSS), 78% LVSI negative or focal, 76% grading 1–2, 94% endometrioid histotype, and 50% without myometrial infiltration. The mean value of neutrophils was 4.79, that of lymphocytes 2.03, monocytes 0.47, eosinophils 0.10, and platelets 258 + 10^3^/mL.

All clinical and parametric characteristics are summarized in Table 1.

**Table 1:** Patients’ characteristics

Characteristic	*N* = 163^a^
Age	62 (14)
BMI	26 (8)
MSI	
MSI	23 (16%)
MSS	121 (84%)
Missing	19
LVSI	
Neg or focal	127 (78%)
Pos	36 (22%)
Grading	
1	62 (38%)
2	62 (38%)
3	39 (24%)
Histotype	
Endometrioid	151 (94%)
Adenosquamous	1 (0.6%)
Serous	8 (5.0%)
Myometrial infiltration	
No infiltration	82 (50%)
< 50%	58 (36%)
≥ 50%	23 (14%)
FIGO stage	
IA1	91 (58%)
IA2	32 (20%)
IA3	2 (1.3%)
IB	33 (21%)
IC	0 (0%)
Neutrophils	4.79 (2.41)
Lymphocytes	2.03 (1.05)
Monocytes	0.47 (0.23)
Eosinophils	0.10 (0.15)
Platelets	258 (91)

^a^Median, (IQR); *n* (%)

### Outcomes

The main outcome was to compare the mean of NL R, MLR, and PLR in the three myometrial infiltration modalities stratified as no infiltration, < 50%, and ≥ 50%.

NLR showed increasing mean values of 2.07, 2.35, and 2.68 for the three categories, with a statistically significant difference (*p* = 0.033). MLR showed the same increasing trend with values of 0.20, 0.23, and 0.26 (*p* = 0.029). PLR, similarly, showed values of 119, 140, and 146 (*p* = 0.043) for the category “no Infiltration,” < 50%, and ≥ 50%, respectively. These values are summarized in Table 2.

**Table 2:** Myometrial infiltration outcomes

Characteristic	No infiltration, *N* = 82^a^	< 50%, *N* = 58^a^	≥ 50%, *N* = 23^a^	*p*-value^b^
NLR	2.07 (1.15)	2.35 (2.21)	2.68 (4.82)	**0.033**
MLR	0.20 (0.07)	0.23 (0.22)	0.26 (0.26)	**0.029**
PLR	119 (62)	140 (90)	146 (94)	**0.043**

^a^Median (IQR)

^b^Kruskal-Wallis rank sum test

We conducted a second stratification for LVSI-negative or focal versus diffuse patients.

NLR showed a mean of 2.17 in LVSI-negative patients and 3.23 in LVSI-positive patients, with a statistically significant difference (*p* = 0.010). MLR showed a mean of 0.20 vs 0.24 for the two categories without reaching statistical significance (0.054). Finally, PLR showed a statistically significant difference with values of 125 and 141 (*p* = 0.033). The results for the stratification by LVSI are summarized in Table 3.

**Table 3:** LVSI outcomes

Characteristic	LVSI neg or focal, *N* = 127^a^	LVSI diffuse, *N* = 36^a^	*p*-value^b^
NLR	2.17 (1.16)	3.23 (4.62)	**0.010**
MLR	0.20 (0.12)	0.24 (0.24)	0.054
PLR	125 (68)	141 (142)	**0.033**

^a^Median (IQR)

^b^Wilcoxon rank sum test

Finally, the same analyses were repeated by stratifying the sample by grading. In the latter analysis, NLR and MLR failed to achieve a statistically significant difference, while MLR averaged 0.21, 0.25, and 0.28 for grading 1, 2, and 3, respectively (*p* = 0.014) (Table 4).

**Table 4:** Grading outcomes

Characteristic	G1, *N* = 62^a^	G2, *N* = 62^a^	G3, *N* = 39^a^	*p*-value^b^
NLR	2.13 (1.27)	2.34 (2.48)	2.26 (1.27)	0.5
MLR	0.21 (0.07)	0.25 (0.23)	0.28 (0.09)	**0.014**
PLR	127 (64)	143 (75)	128 (63)	0.3

^a^Median (IQR)

^b^Kruskal-Wallis rank sum test

**Fig. 1:**
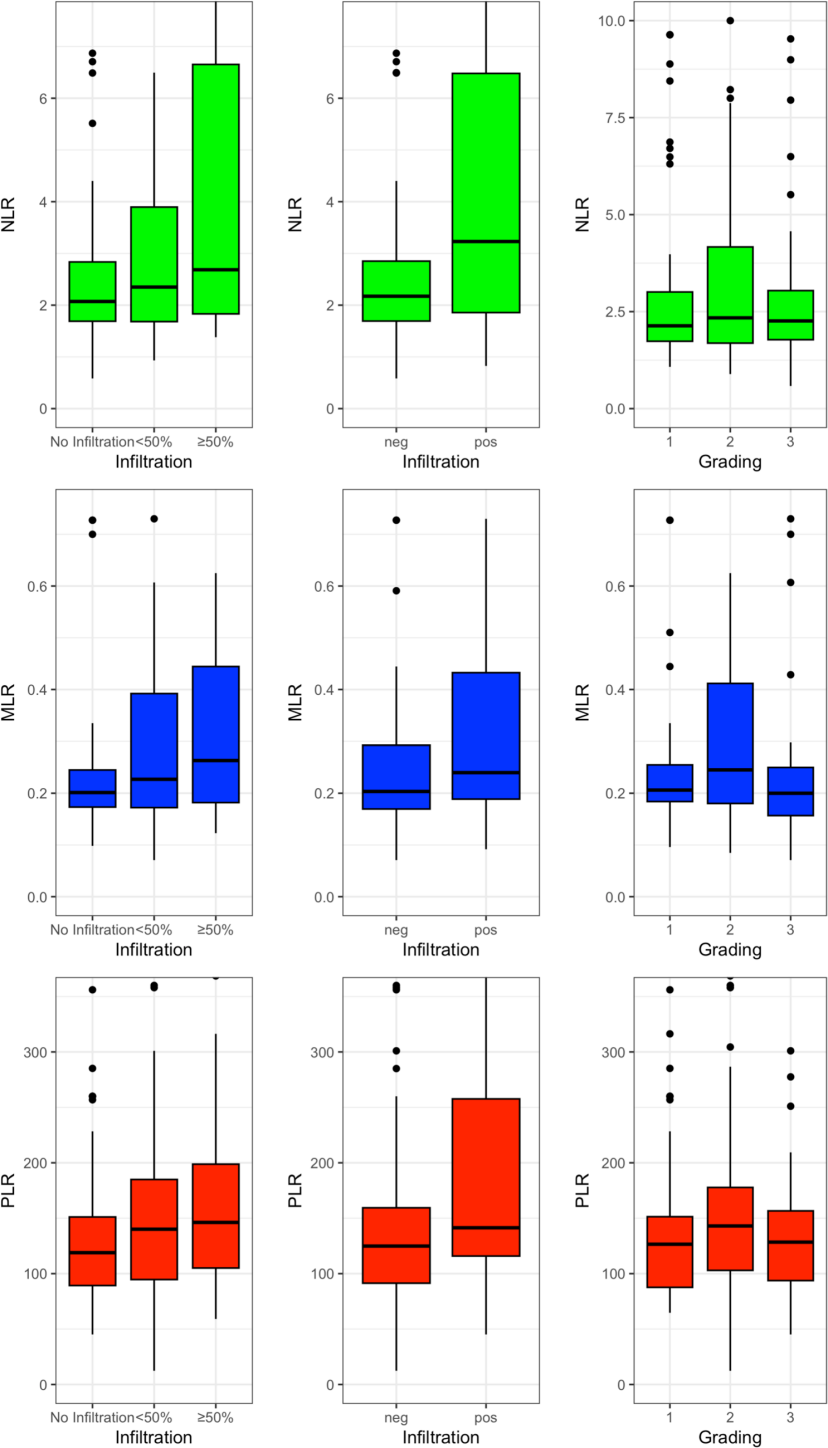
Boxplot

The distribution of the analyzed parameters is represented by box plots shown in Fig. 1.

#### Linear Regression

To assess the correlation between the histopathological parameters taken into account and the indices of inflammation, we constructed a linear regression model including the three parameters (myometrial infiltration, LSVI, grading). The analysis showed a statistically significant relationship coefficient only between myometrial infiltration and MLR (beta 0.07, CI 95% 0.01–0.13, *p* = 0.041) (Table 5).

**Table 5:** Multivariate linear regression

Characteristic	NLR			MLR			PLR		
	Beta	95% CI^a^	*p*-value	Beta	95% CI^a^	*p*-value	Beta	95% CI^a^	*p*-value
LVSI									
Neg or focal	—	—		—	—		—	—	
Diffuse	0.16	− 1.7, 2.1	0.9	0.03	− 0.08, 0.13	0.6	21	− 15, 58	0.3
No myometrial infiltration	—	—		—	—		—	—	
Any myometrial infiltration	1.1	− 0.03, 2.3	0.056	0.07	0.01, 0.13	**0.041**	16	− 6.2, 38	0.2
Grading									
1	—	—		—	—		—	—	
2	0.84	− 1.0, 2.7	0.4	0.07	− 0.03, 0.17	0.2	10	− 26, 46	0.6
3	− 0.67	− 2.5, 1.1	0.5	− 0.05	− 0.15, 0.04	0.3	− 3.5	− 38, 31	0.8

^a^*CI* confidence interval

## Discussion

### Results Interpretation

The study shows additional prognostic factors in the early stages of endometrial carcinoma correspond to an alteration of the inflammation indices for all parameters considered. This may be justified because parameters such as myometrial infiltration and LVSI positivity represent a point of contact between the tumor environment and the patient, thus triggering an inflammatory response from the patient [20]. This principle is less intuitive concerning grading, which does not represent a “territorial extension” of the tumor but its histological degeneration [21, 22]. The distribution of the averages of the phlogosis indices was less preponderant when compared to this last parameter. The multivariate analysis finally created a regression model that took into account all three parameters analyzed, showing that the greatest weight on the inflammatory response is exerted by myometrial infiltration. Myometrial infiltration is indeed a well-known independent prognostic factor. It manifests natural tumor progression, deepening locally before facilitating distant metastatisation [23]. It is no coincidence that the recent update of the FIGO staging in 2023 reintroduced the concept of intramucosal carcinoma, emphasizing the time the carcinoma starts its myometrial infiltration as crucial [24, 25]. This factor in the multivariate analysis probably incorporated the weight exerted by LVSI and grading. Nevertheless, the big difference in the averages reported for all three indices of inflammation examined in patients with and without infiltration of the lymphovascular spaces highlights how this documented progression at the microscopic level also results in a response by the body. The same argument would seem to apply to grading; however, multivariate regression did not show a statistically significant association, but only a trend for patients with grading 3.

Finally, the relationship between inflammation and histopathological parameters can be interpreted in a bivalent manner: the progression of the microscopic tumor can influence the subject’s inflammatory response, and the inflammatory state can promote its progression [26].

### Clinical Implications

Indices of inflammation are clinical parameters easily obtained during the diagnostic framing of endometrial carcinoma [4, 5]. Knowing their relationship with prognostic factors can help personalize counseling, diagnostic, and therapeutic courses. For example, the presence or absence of myometrial infiltration is the pivotal factor in subjecting a patient desiring offspring to fertility-sparing treatment (FST) [27]. To date, myometrial infiltration is presumed by transvaginal ultrasound and confirmed by the gold standard examination, which remains histopathological analysis. This is, however, precluded in the case of FST [28]. A second-level investigation is represented by nuclear magnetic resonance imaging (MRI) [29]. Evidence of alterations in inflammation indices can help better select patients amenable to this type of investigation. On the other hand, the histological diagnosis of endometrial carcinoma is often made following hysteroscopic biopsies. Although a high concordance rate between biopsy and post-surgical histological diagnosis is reported in the literature, in a percentage of cases the bishop, the biopsy may not be representative of the whole tumor estimating parameters such as grading and LVSI [30]. LVSI may be difficult to obtain at the diagnostic stage, especially when the histological specimen is obtained by curettage [31]. In this scenario, indices of inflammation require an additional evaluation by the clinician to minimize this error threshold. In the same way, precedent studies have shown how alterations in indices of inflammation can predict myometrial infiltration in endometrial carcinomas [32] and help in the differential diagnosis of atypical endometrial hyperplasia [33]. This could also be particularly relevant in directing clinical choices in fertility-sparing treatments.

### Strength and Limitations

In our opinion, the strength of this study is its prospective nature. This nature was necessary to carefully select patients with no confounders regarding pro-inflammatory status. In fact, excluding patients with chronic inflammatory and autoimmune-based systemic diseases made it possible to examine a population with no factors external to the endometrial tumor that could lead to an inflammatory response. The main limitation is the absence of follow-up, which deprives the study of a prognostic evaluation. Although those considerations are not part of the study’s objectives, we cannot exclude the possibility that pathologies with a greater propensity to recurrence may disturb the systemic inflammatory status. Furthermore, a second limitation is that the study was conducted in the same geographical area, making it impossible to exclude the influence of environmental factors. Both these limitations can be overcome by extending the enrolment over time and enrolling centers.

## Conclusion

In stage I endometrial carcinomas, the presence of risk factors such as LVSI, myometrial infiltration, and grading 3 is associated with higher mean values of NLR, MLR, and PLR. However, a multivariate analysis showed that the strongest correlation occurs with myometrial infiltration. Further studies will be necessary to make prognostic sense of these results.

## Data Availability

All data and the methodological process for their calculation can be supplied under explicit request to the corresponding author and provided as an ‘.R’ file.

## Declarations

### Generative AI in Scientific Writing

The authors declare that no AI was used to write the original draft. Grammar correction tools (Grammarly, Inc.) were used to improve the quality of English and readability. The technology was used under human oversight and control.
